# The complete mitochondrial genome of Siberian roe deer (*Capreolus pygargus bedfordi*) and its phylogenetic analysis

**DOI:** 10.1080/23802359.2019.1711232

**Published:** 2020-02-13

**Authors:** Danyi Ao, Yongfang Yao, Diyan Li, Meng Xie, Qingyong Ni, Mingwang Zhang, Huailiang Xu

**Affiliations:** a College of Life Science, Sichuan Agricultural University, Ya’an, Sichuan, China;; b College of Animal Science and Technology, Sichuan Agricultural University, Chengdu, Sichuan, China

**Keywords:** Mitochondrial genome, *Capreolus pygargus bedfordi*, phylogenetic analysis

## Abstract

Due to the multiple causes, the population of roe deer has declined significantly. In this study, we analyzed the complete mitogenome of *Capreolus pygargus bedfordi*, whose genome was 16,357 bp long. There were 13 protein-coding genes (PCG), two ribosomal RNA genes (*12S rRNA* and *16S rRNA*), 22 transfer RNA genes, and one control region. Nine PCGs started with ATG, while *NAD2*, *NAD3*, and *NAD5* genes commenced with ATA, and *ND4L* began with GTG. *ND6* and eight tRNA genes were encoded on the L-strand. These results provide newer molecular information, which contribute to its molecular and phylogenetic studies, and genetic diversity conservation.

The roe deer (*Capreolus*) belongs to Cervidae, including two species: the European roe deer (*Capreolus capreolus*) and the Siberian roe deer (*Capreolus pygargus*). The Chinese roe deer belongs to Siberian roe deer, which has two subspecies: *Capreolus pygargus pygargus* and *Capreolus pygargus bedfordi* (Hewison and Danilkin 2001; Smith et al. 2010). They are widely distributed in central, southwest, northwest, and northeast China. Roe deer is one of the important food sources for many raptors (Jiang et al. 2015). Therefore, studying it could provide more information about the protection of raptor populations. In addition, Siberian roe deer is listed as Least Concern (LC) in the Red List by International Union for the Conservation of Nature (IUCN) (Lovari et al. 2016). In this study, we analyzed the complete mitogenome of *C. p. bedfordi*, which could provide more information for further molecular and phylogenetic studies, and genetic diversity conservation.

The muscle tissue of the roe deer was collected from illegal poaching dead individuals captured by forest police in Nanjiang, Sichuan of China (106°83′E, 32°35′N) and stored in Sichuan Agricultural University Museum (number 000758). The total genomic DNA was extracted by the standard phenol–chloroform method (Sambrook et al. 1990). The sequence of *Capreolus pygargus tianschanicus* (NC039093) was downloaded from NCBI as the reference sequence to design primers (13 pairs). All primers were used to amplify the complete mitogenome of roe deer. Detecting products by gel electrophoresis and then bidirectionally sequenced those products. Sequences were edited and compared by SeqMan Software (Siasi et al. 2012). The phylogenetic tree was constructed in MEGA6 (Tamura et al. 2013). Transfer RNA genes were predicted by ARWEN (Laslett and Canback 2008).

The total length of the complete mitogenome sequence was 16,357 bp (GenBank accession number MN813763). There were 13 protein-coding genes (PPG), two ribosomal RNA genes (*12S rRNA* and *16S rRNA*), 22 transfer RNA genes and 1control region (D-loop). The base composition of the sequence included 33.5% A, 30.02% T, 23.14% C, 13.34% G with an apparently AT biased (63.52%). For all protein-coding genes (PCGs), 5 (*COX3*, *ND1–4*) had incomplete stop codons, 8 had complete stop codons, of which *ATP8* ended with TAG, *CYTB* with AGA, and the remaining 6 with TAA. Nine PCGs started with ATG, while *NAD2*, *NAD3*, and *NAD5* genes commenced with ATA, and *ND4L* began with GTG. *ND6* and eight tRNA genes were encoded on the L-strand while others on the H-strand. The lengths of *12S rRNA* and *16S rRNA* were 955 and 1569 bp, respectively, and separated by the *tRNA-Val* gene. In the end, the D-loop was 1143 bp in length.

The complete mitogenome of *C. p. bedfordi* and other 15 species, which are from Cervidae and one outgroup (*Gazella subgutturosa*) were used to establish a maximum-likelihood (ML) phylogenetic tree in MEGA6 (Tamura et al. 2013; Figure 1). The ML tree showed that our sequence with *C. p. tianschanicus* formed a strongly supported (100% bootstrap) sister branch. This result provides more molecular and evolutionary information about this species.

**Figure 1.**
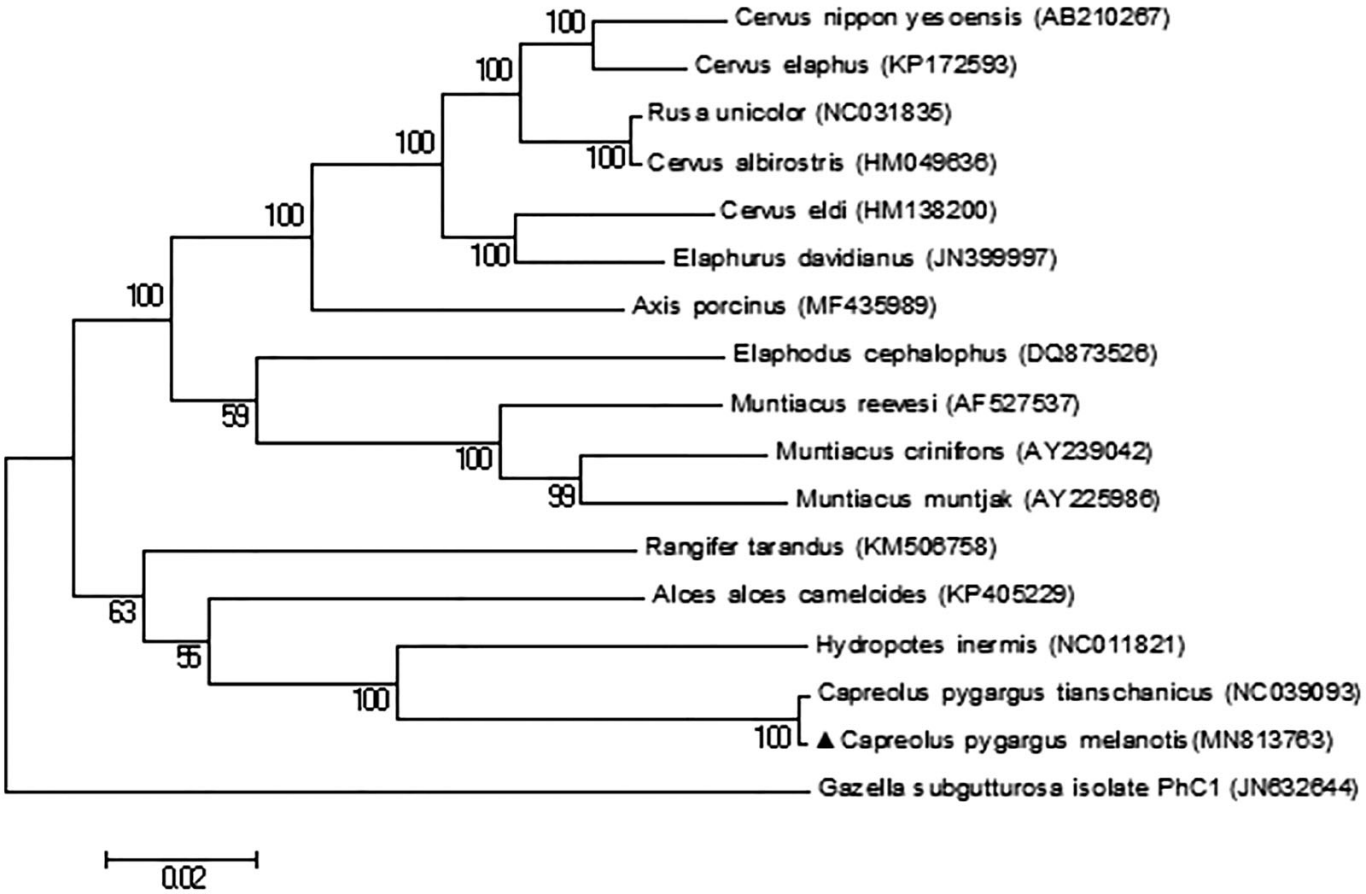
Maximum-likehood (ML) phylogenetic tree constructed based on a complete mitogenome of *C. p. bedfordi* and the other 15 species from Cervidae and one outgroup (*Gazella subgutturosa*). Numbers at the branches refer the bootstrap values with 1000 replications. Solid triangle represents the sequence in this study.
